# Morphometric, Biomechanical and Macromolecular Performances of β-TCP Macro/Micro-Porous Lattice Scaffolds Fabricated via Lithography-Based Ceramic Manufacturing for Jawbone Engineering

**DOI:** 10.3390/jfb16070237

**Published:** 2025-06-28

**Authors:** Carlo Mangano, Nicole Riberti, Giulia Orilisi, Simona Tecco, Michele Furlani, Christian Giommi, Paolo Mengucci, Elisabetta Giorgini, Alessandra Giuliani

**Affiliations:** 1 Department of Dental Sciences, University Vita Salute San Raffaele, 20132 Milan, Italy; camangan@gmail.com; 2 Department of Clinical and Molecular Sciences, Marche Polytechnic University, 60126 Ancona, Italy; n.riberti@staff.univpm.it; 3 Department of Clinical Specialistic and Dental Sciences, Marche Polytechnic University, 60126 Ancona, Italy; g.orilisi@univpm.it; 4 I.R.C.C.S. San Raffaele Hospital, University Vita Salute San Raffaele, 20132 Milan, Italy; tecco.simona@hsr.it; 5 Department of Biomedical Sciences and Public Health, Marche Polytechnic University, 60126 Ancona, Italy; 6 Department of Life and Environmental Science, Marche Polytechnic University, 60131 Ancona, Italy; c.giommi@pm.univpm.it (C.G.); e.giorgini@staff.univpm.it (E.G.); 7 Department Science and Engineering of Materials, Environment and Urban Planning, Marche Polytechnic University, 60131 Ancona, Italy; p.mengucci@staff.univpm.it

**Keywords:** β-TCP, lithography-based ceramic 3D printing, bone regeneration, scaffold, jawbone engineering, maxillary sinus augmentation, microarchitecture, osteocyte lacunae network

## Abstract

Effective bone tissue regeneration remains pivotal in implant dentistry, particularly for edentulous patients with compromised alveolar bone due to atrophy and sinus pneumatization. Biomaterials are essential for promoting regenerative processes by supporting cellular recruitment, vascularization, and osteogenesis. This study presents the development and characterization of a novel lithography-printed ceramic β-TCP scaffold, with a macro/micro-porous lattice, engineered to optimize osteoconduction and mechanical stability. Morphological, structural, and biomechanical assessments confirmed a reproducible microarchitecture with suitable porosity and load-bearing capacity. The scaffold was also employed for maxillary sinus augmentation, with postoperative evaluation using micro computed tomography, synchrotron imaging, histology, and Fourier Transform Infrared Imaging analysis, demonstrating active bone regeneration, scaffold resorption, and formation of mineralized tissue. Advanced imaging supported by deep learning tools revealed a well-organized osteocyte network and high-quality bone, underscoring the scaffold’s biocompatibility and osteoconductive efficacy. These findings support the application of these 3D-printed β-TCP scaffolds in regenerative dental medicine, facilitating tissue regeneration in complex jawbone deficiencies.

## 1. Introduction

Rehabilitation of edentulous patients with dental implants can be challenging due to insufficient alveolar bone volume resulting from alveolar atrophy and maxillary sinus pneumatization. In such anatomical conditions, achieving primary stability is often difficult owing to the lack of adequate cortical bone. Biomaterials can augment the body’s innate capacity for bone regeneration, as newly forming bone requires a scaffold to facilitate and support the regenerative process.

Various biomaterials have been investigated for use in bone regeneration procedures, including demineralized freeze-dried bone allografts, calcium carbonate, bioactive glass, polymers such as polylactic acid (PLA) and polyglycolic acid (PGA), bovine-derived xenografts and peptides, calcium sulfate, bovine deproteinized bone, and hydroxyapatite. Although autologous bone remains the gold standard due to its superior osteogenic potential, constraints including donor site morbidity, limited availability and the need for additional surgical procedures pose significant disadvantages. Consequently, synthetic biomaterials have been developed to overcome these limitations [1]. In particular, concerns regarding disease transmission associated with allografts and xenografts have driven research toward synthetic bone substitutes that aim to replicate the physical and chemical properties of native bone tissue, with the goal of achieving osteoconduction, osteoinduction, and osteointegration [2,3].

An ideal biomaterial should possess specific biological and clinical characteristics. Biologically, it should facilitate mesenchymal cell recruitment via host-derived growth factors and exert bioactive effects to promote ossification. Additionally, it should provide a three-dimensional scaffold supporting vascular ingrowth and osteoprogenitor cell migration and be bioresorbable over time. Clinically, the material should be user-friendly, cost-effective, and radiographically evaluable throughout the healing process. To facilitate monitoring of resorption and substitution, the biomaterial should be radiopaque.

Bone engineering within a scaffold involves cellular recruitment, infiltration from surrounding bone tissue, and vascularization [4,5]. High porosity has been shown to enhance osteogenesis, as confirmed by numerous studies. Bioceramics that mimic natural bone can combine favorable mechanical properties with an interconnected porous architecture, making them suitable as delivery vehicles for cells. Structurally, pores larger than 100–150 µm are necessary to ensure adequate vascularization and tissue ingrowth [6]. Moreover, an optimal biomaterial should degrade gradually, being fully replaced by vital bone tissue, with the resorption rate matched to the rate of new bone formation; excessively rapid degradation can impair regenerative outcomes [7,8].

Rapid Prototyping (RP) is an additive manufacturing technology that constructs three-dimensional structures through layer-by-layer deposition of materials. One significant advantage of RP techniques is their capacity to produce patient-specific scaffolds based on cone-beam computed tomography (CBCT) data, which is particularly beneficial for complex defect geometries [9]. Lithography-based ceramic manufacturing (LCM) is an additive manufacturing process that uses a photopolymerization technique to create ceramic parts. It involves building up layers of a UV-curable ceramic suspension (slurry) via photolithography, followed by debinding and sintering to form a dense ceramic object. This method allows for the fabrication of complex geometries that are difficult to achieve with traditional ceramic manufacturing processes. LCM offers an excellent approach for fabricating bone substitute scaffolds that replicate biomimetic architectures with optimal interconnected macro-porosities [10]. The microenvironment can be further optimized through control of sintering parameters. This LCM process opens new avenues for the shaping of ceramic scaffolds: it not only utilizes the same ceramic material as traditional production methods and is capable of producing components composed of 100% ceramic material despite the presence of photopolymers during the process, but also ensures that both precision and density are maintained while exhibiting mechanical properties comparable to conventionally fabricated parts. By employing this approach, it is possible to significantly reduce the time-to-market, thereby supporting the goals of personalized medicine. This technique has already been tested in a case report involving alveolar ridge augmentation with Hydroxyapatite (HA) scaffolds [11] and in a clinical trial for the rehabilitation of the atrophic maxilla [12], although the characterization of the β-TCP ceramic block grafts used in these studies was not performed.

In this context, recent interest has been directed towards β-tricalcium phosphate (β-TCP) printed scaffolds: their biodegradable nature enables them to serve as temporary bone substitutes, providing mechanical support and facilitating biological activities during tissue regeneration [8,13]. Various recent approaches for fabricating porous β-TCP ceramic scaffolds with micro-porous struts have been proposed [14]; however, some methods are significantly influenced by the mechanical properties of porous β-TCP scaffolds with non-uniform pore sizes. Additive manufacturing techniques, such as 3D printing, have been suggested as promising strategies to produce highly porous β-TCP scaffolds with optimized pore size, porosity, and mechanical characteristics. A patient-specific β-TCP scaffold for alveolar ridge augmentation has been explored in a case report; however, no information has been reported on how the scaffold was produced [15]. Indeed, a rigorous characterization of the LCM-printed β-TCP scaffolds is currently missing in the literature.

Extensive research indicates that the three-dimensional optimization of macro- and micro-structures enhances the bioactivity of bone graft substitutes and is critical for successful bone tissue engineering. While the CAD drawings define the scaffold macro-porosity, the printing parameters was proved to totally determine the microporosity of the scaffold [16]. Macro-porosities with pore sizes between 300 and 700 µm are essential for neovascularization and new bone formation without compromising mechanical integrity. An interconnected pore network is vital for cellular infiltration, while microporosity and surface micro-roughness positively influence osteoblast adhesion, proliferation, and differentiation [17].

This multidisciplinary experimental study aimed to verify the morphometric, biomechanical and macromolecular performances of a new β-TCP scaffold for jawbone engineering, fabricated with an innovative macro/micro-porous lattice via lithography-based ceramic manufacturing. The performance of the scaffolds was evaluated in comparison to data from previous studies and native bone. Mechanical and biological assessments offered valuable insights for optimizing scaffold design to enhance bone regeneration efforts.

## 2. Materials and Methods

### 2.1. Design and Production

Nine (*n* = 9) cubic TCP scaffolds were fabricated using a TCP slurry (LithaBone™ TCP 300, Lithoz, Vienna, Austria), as previously described [18]. Briefly, the scaffolds were produced via additive manufacturing using the CeraFab 7500 system (Lithoz, Vienna, Austria), which selectively solidifies the slurry by photopolymerization. Each 25 µm layer of a photoactive polymer was exposed to blue LED light at a resolution of 50 µm in the x/y-plane. The green body of the scaffold was built in a layer-by-layer manner [19]. Following fabrication, the green body was carefully detached from the build platform using a razor blade, and then cleaned with LithaSol 20™ (Lithoz, Vienna, Austria) and pressurized air to remove residual powder.

The polymeric binder, responsible for binding the ceramic particles, was decomposed through a thermal treatment regime, followed by sintering to densify the ceramic structure. The sintering process involved a dwell time exceeding >48 h at temperatures of >1000 °C, allowing for controlled microporosity via partial sintering. Variations in green body shrinkage due to different maximum sintering temperatures were compensated by dimensional adjustments in all three axes, ensuring that the final macro- and microarchitecture remained consistent post-sintering. The sintered scaffolds were packaged for integration into the surgical workflow and utilized as bone substitute implants with gamma sterilization.

**Figure 1:**
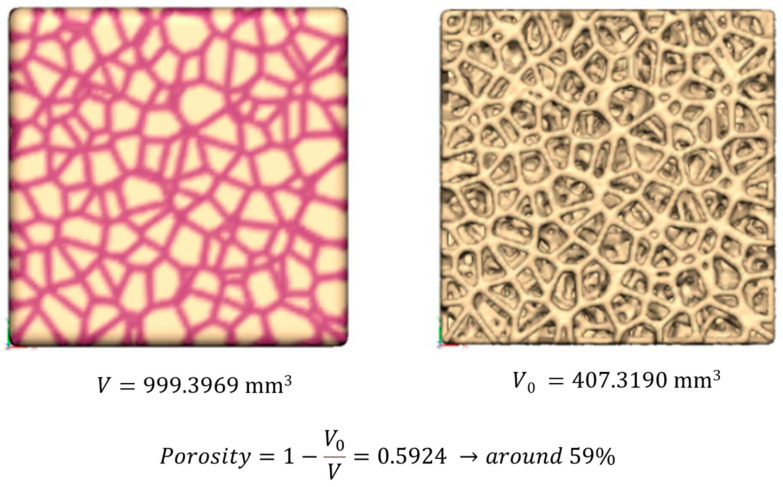
Nominal scaffold geometry: indication of the nominal overall volume (V), actual TCP volume (V_0_), and porosity percentage (%).

The scaffold geometry, with indication of the nominal overall volume (V), actual TCP volume (V_0_), and porosity percentage (%), is reported in Figure 1.

### 2.2. Clinical Case Description

A 60-year-old male patient required implant-supported prosthetic rehabilitation of the left posterior maxillary region. Cone-beam computed tomography (CBCT) revealed significant alveolar bone atrophy, necessitating maxillary sinus floor elevation to facilitate implant placement. During the initial consultation, comprehensive clinical and occlusal examinations were conducted, along with periapical radiographs and CBCT imaging.

A routinary procedure of maxillary sinus augmentation was performed in the atrophic posterior maxilla utilizing a three-dimensionally printed β-tricalcium phosphate (β-TCP) graft (KLS Martin SE & Co. KG, Mühlheim an der Donau, Germany). The sinus augmentation procedure adhered to the classical lateral window technique as described by Tatum OH (1986) [20].

The surgical approach involved a horizontal crestal incision combined with two vertical releasing incisions extending beyond the mucogingival junction, followed by reflection of a full-thickness mucoperiosteal flap to expose the lateral wall of the maxillary sinus. An osteotomy of approximately 1.0 ± 0.1 cm was delineated and isolated using piezo surgical instruments. The bony window was carefully removed and preserved in a sterile saline solution. The Schneiderian membrane was then meticulously elevated and protected with dedicated elevators to prevent perforation.

A block of 3D-printed β-TCP, fabricated via the same additive manufacturing process described previously, was inserted into the sinus cavity to serve as the graft material. Following graft placement, the bony window was repositioned to restore the lateral sinus wall, and the surgical site was closed with sutures to ensure complete flap adaptation. Postoperative antibiotic therapy was administered.

After a healing period of six months, a secondary surgical procedure was performed for re-entry. During this classical routinary procedure, a 10 mm bone core is normally harvested (biopsy) using a trephine bur (diameter 2.5 mm, length 15 mm) under copious saline irrigation to insert a dental implant (Megagen Implant Co., Gyeongbuk, Republic of Korea). So, in this case, a dental implant was placed precisely at the site of the biopsy, guided by a cone-beam computed tomography (CBCT) template for guided bone regeneration. Three months following implant placement, definitive prosthetic rehabilitation was completed with zirconia-ceramic fixed prostheses.

The patient gave his informed consent to use the harvested bone for observational analyses (Macromolecular Fourier Transform InfraRed Imaging; histology; X-ray micro-computed tomography, Phase-contrast X-ray micro-computed tomography; Convolutional Neural Network for Osteocyte Lacunae Analysis; Bone Mineralization Analysis).

In addition, the preliminary in vitro results obtained by mechanical tests of scaffolds and by X-ray exams of scaffolds (mechanical tests, SEM analysis, X-ray diffraction, X-ray micro-computed tomography) were also retrospectively analyzed.

The protocol was approved by the Ethical Committee “Comitato Etico Territoriale Lombardia 1”, Milan, Italy (CET 462-2024, 9 December 2024).

### 2.3. Mechanical Tests

Compressive tests were conducted on all the scaffolds in a continuous mode until fracture, utilizing a Material Testing Stage (MTS1) integrated within the Bruker Sky-Scan 1174 experimental chamber, replacing the standard sample holder. Throughout the testing process, the scaffolds were subjected to load application controlled by specialized software; the corresponding load–deformation curve was displayed in real time on the screen, with the deformation measured accurately by a high-precision displacement sensor. The MST1 stage has the following characteristics: maximum force: 440 N; displacement sensor accuracy: ±0.01 mm; load measurement accuracy = ±4 N (±1% of the full range); maximum object diameter: 20 mm; maximum object height (in compression mode): 23 mm.

All β-TCP cubic scaffolds were submitted to compressive tests: *n* = 4 samples were loaded in the direction of lithography-based manufacturing, while the remaining *n* = 5 samples were loaded in a random direction during this test.

### 2.4. Structural and Morphological Characterization

#### 2.4.1. SEM Analysis

Scanning electron microscope (SEM) observations were performed by Tescan Vega 3 (Tescan Company, Brno, Czech Republic) in order to observe the β-TCP scaffold surface and inner structure, before and after fracture due to compression loading.

#### 2.4.2. X-Ray Diffraction

Structural characterization of β-TCP raw powder and scaffolds was carried out by X-ray diffraction (XRD) using a Bruker D8 Advance diffractometer (Billerica, MA, USA) operating at V = 40 kV and I = 40 mA, with Cu-Kα radiation, in the angular range 2θ = 5–80°. Pattern analysis was performed by DIFFRAC. EVA (Version 7, provided by Bruker, Billerica, MA, USA)) software with the PDF2 database of the International Centre for Diffraction Data (ICDD). Rietveld refinement of XRD patterns was carried out by the ReX Powder Diffraction software, version 0.9.4 [21] after calibration of the instrumental broadening by reference Al_2_O_3_ powder. The crystallographic structure of the Ca_3_(PO_4_)_2_ (Whitlockite) phase with nominal lattice parameters a = 1.0429 nm and c = 3.7380 nm (ICDD file n. 9-169) used in Rietveld refinement was obtained from the Crystallography Open Database (COD, http://www.crystallography.net/cod/, accessed on 22 April 2025).

#### 2.4.3. X-Ray Micro-Computed Tomography (XμCT)

The nine scaffolds were investigated by X-ray micro-computed tomography (XμCT), using a desktop device Bruker SkyScan 1174 μ-CT system (SkyScan-Bruker, Antwerp, Belgium). The projection settings used were the following: acceleration voltage of 50 kV; beam current of 800 μA; aluminum filter (thickness of 1 mm); pixel size equal to 11.5 μm; rotation of 180° in steps of 0.2°; exposure time per projection equal to 10 s. On average, the scanning process took about 3 h. To convert the projections into transversal slices, NRecon software (version 1.6.10.2, Bruker, Billerica, MA, USA) was used, employing the following correction settings: ring artifact reduction (3); smoothing (5); beam hardening (50%).

The 3D microstructural analysis was carried out using the BoneJ plugin within FIJI [22] to evaluate the following morphological parameters: the BV/TV specific volume (Vol.%), measuring the overall mineralized (β-TCP) volumetric density, i.e., the ratio between the mineralized volume and the total volume of the scaffold; the mean trabecular thickness (Tb.Th; μm) and the mean trabecular spacing (Tb.Sp; μm), measuring the mean distance between two trabeculae. The 3D orientation and arrangement indices (namely, the Connectivity Density (Conn.D; px^−3^), the Anisotropy Degree (DA), and the Fractal Dimension (FD)) were also calculated. The Conn.D parameter is designed to estimate the number of connected structures, i.e., trabeculae in a network. This connectivity measurement is related to a topological number χ known as Euler number. Mathematically, connectivity is defined = 1 − (χ + Δχ), where χ describes the shape or structure of a topological space and the term Δχ corrects for the change in the topology of an object, when it is cut to pieces; the input image must be 3D and binary; the resulting parameter is the Connectivity density (Conn.D): number of elements per unit volume, showing higher values for better-connected trabeculae and lower values for poorly connected ones. DA was used to quantify the directionality of the trabeculae; it evaluates whether the trabeculae have a certain orientation, or if they are randomly aligned. DA = 0.0 means that the structure is completely isotropic; thus, the trabeculae have no preferential directionality; DA = 1.0 means that there is a prevailing orientation in the structure of the scaffold. The fractal dimension (FD) is a statistical measure that quantifies the complexity of the trabecular pattern. It is determined from a series of images using the box-counting method, where a grid of decreasing size is overlaid on the image, and the number of boxes that contain at least one foreground voxel is tallied. As the box size becomes smaller and the grid becomes more detailed, the number of boxes covering the structure increases in a fractal manner. In three dimensions, the FD value ranges from 2, indicating a planar (2D) distribution, to 3, representing a fully three-dimensional structure. The box-counting algorithm was configured with the following initial parameters [23]: box initial size [px]: 48; smallest box size [px]: 6; box scale factor: 1.2; grid translation: 0.

Three of the previous nine scaffolds were retested by XμCT after the compressive load test, using the same experimental parameters. The same morphometric parameters previously described were also calculated on these samples, with the exclusion of the Tb.Sp, unreliable after the sample fracture.

The same XμCT (SkyScan-Bruker, Antwerp, Belgium) device was used to investigate the patient’s biopsy using the following experimental settings: acceleration voltage of 50 kV; beam current of 800 μA; aluminum filter (thickness of 1 mm); pixel size equal to 6.5 μm; rotation of 180° in steps of 0.4°; exposure time per projection equal to 11 s. The 3D volume reconstruction was achieved employing the following correction settings: ring artifact reduction (3); smoothing (3); beam hardening (30%). In this biopsy, the same morphometric parameters investigated in the as-produced scaffolds were analyzed. In addition, the percentage ratios between bone/biomaterial in the process of ossification/residual biomaterial was quantified.

#### 2.4.4. Phase-Contrast X-Ray Micro-Computed Tomography (PhC-XμCT)

Phase-contrast X-ray micro-computed tomography (PhC-XμCT) was carried out at the SYRMEP beamline of the ELETTRA Synchrotron Radiation facility (Trieste, Italy). An analysis was performed on the patient’s biopsy already investigated by desktop XµCT to obtain morphometric information on the osteocyte lacunae network and on the newly formed bone mineralization distribution. The method used for PhC-XμCT measurements differs from the XμCT analysis used to assess scaffold morphometric parameters. In standard XμCT, the imaging relies on differences in absorption contrast, whereas in this specific synchrotron phase-contrast setup, the imaging is based on phase shifts of the X-ray wave as it interacts with the sample. For X-rays, the refractive index n of the material is a complex number equal to *n* = 1 − δ + iβ. In this equation, δ is the decrement of the real part of the complex refractive index n, while the imaginary part β describes the material absorption. For peak energies used to investigate biological tissues, δ is about three orders of magnitude greater than β, thus resulting in higher sensitivity of the phase-contrast approach respect to absorption contrast.

The experiment was set with the following parameters: the imaging system captured a total of 1800 projections over 360° sample rotation, with exposure time per projection of 450 ms; the white beam was filtered with 1 mm thick silicon (Si) plate leading to a peak energy of 19.3 keV; sample-to-detector distance was set to 100 mm in order to enable phase contrast imaging. The raw images were collected by using an Orca Flash 4.0 CMOS camera (2048 × 2048 pixels, physical pixel size 6.5 μm × 6.5 μm) coupled with a 17 μm GGG scintillator screen. Owing to the optical magnification system used in combination with the detector, the effective pixel size selected for imaging was 0.9 μm × 0.9 μm, yielding a field of view of 1.8 mm × 1.8 mm.

After the acquisition, the patient’s biopsy was reconstructed using the STP software (SYRMEP Tomo Project, v.112022a) [24]. The parameters selected for the slice reconstruction were Rivers ring removal (wd = 3) for ring removal correction, Paganin TIE-Hom algorithm for the phase retrieval step [25], and the Filtered Back Projection algorithm, in combination with the Shepp-Logan filter, for the ultimate reconstruction step. In detail, phase retrieval was performed by applying Paganin’s algorithm with a fixed δ/β ratio of 100. Finally, the reconstructed stack comprising 2048 images was converted in 8-bit images.

#### 2.4.5. Convolutional Neural Network for Osteocyte Lacunae Analysis

The image processing was conducted by means of the Dragonfly software [26] (Dragonfly 2022.2, Comet Technologies Canada Inc., Montreal, QC, Canada), exploiting a deep learning tool which applies artificial intelligence to the segmentation process. Moreover, the Scalar Generator tool was used to extract quantitative information on eight specific regions of interest (ROIs—box dimension: 534 × 534 × 534 μm^3^) virtually extracted from the patient biopsy. In particular, two sub-volumes were extracted from both the apical and coronal portions, and four sub-volumes were extracted from the central portion.

The network employed on these portions of bone had already been previously trained and applied [27]. In order to detect osteocyte lacunae and discriminate them from the bone matrix and the background, a Pre-Trained U-Net was chosen (one of the AI tools of the Dragonfly software), and hyperparameters (Table S1) were set for the training phase. The use of this neural network model enabled the precise detection of the osteocyte lacunae in an automatic way and short time [28], mapping the full biopsy.

Class count refers to the number of components that the network must discriminate; the depth level is the number of layers that capture contextual information in the two phases of encoding and decoding; the initial filter count represents the filter count at the first convolutional layer; the patch size is the number of sub-sections in which the dataset will be cut; the stride ratio specifies the overlap between adjacent patches; the batch size determines the number of patches in a batch; the epoch’s number indicates the number of iterations; the loss function measures the error between the neural network’s prediction and reality; the optimization algorithm is used to update the parameters of the model, to minimize the prediction errors; the metric function is used to judge the model’s performance; early stopping is used to stop iterations once the estimation error drops below a certain threshold; and the learning rate is a parameter which controls the step with which we arrive at the final network optimization result. Generally, this last parameter reduces as one becomes closer to optimal performance; we chose to have it adapted during training and not to keep it fixed. The percentage division of the dataset is 90% for training and 10% for testing.

Network performance was evaluated by applying the training to each ROI and visually verifying that it accurately segmented each osteocyte lacuna. The region of interest (ROI) of the lacunae selected and the Scalar Generator tool was applied; it considered each lacuna individually; afterwords, morphometric parameters were measured in each osteocyte lacuna.

According to the literature [29,30], only lacunae within the volumetric range of (80–980) µm^3^ can be considered functional; thus, only lacunae in this volumetric range were considered.

The following shape complexity features (SCFs) were evaluated in each osteocyte lacuna to quantify the degree of detail or complexity that its shape possesses: the mean lacuna volume (LacV—μm^3^); the mean lacunar volume/surface ratio (LacV/S—µm) [31]; the 3D aspect ratio (LacAR), which returns the ratio between the lacuna shortest and longest axes; the Sphericity (LacS), which quantifies the extent to which the lacuna resembles a sphere by calculating the ratio of the surface area of an equal-volume sphere to the actual surface area of the lacuna; and the specific number of lacunae (LacNr—mm^−3^), which refers to the number of lacunae within a 1 mm^3^ volume of bone matrix.

#### 2.4.6. Bone Mineralization Analysis

Nine specific regions of interest (ROIs—box dimension: 600 × 600 × 600 pixel^3^), i.e., three for each axial (coronal, central, apical) portions, were virtually extracted from the patient biopsy.

Starting from the reconstructed complex refractive index n that is linearly related to the mass density (mg/cm^3^), the apparent bone mineralization distribution (BMD^r^) was calculated in each ROI of the biopsy, following the Roschger approach [32]. The BMD^r^ parameters were calculated within the mineralized domain; since the reconstructed complex refractive index might be biased due to the constant ratio δ/β and the use of a white beam, absolute values of bone mass density (calcium concentrations − Ca weight %) could not be retrieved; hereafter, the superscript r denotes relative values for all the parameters. Indeed, the Paganin phase retrieval algorithm [25] is based on the assumption of a monochromatic beam and works as an approximation in the present analysis. However, since ROIs were similar in terms of size and composition, relative differences in bone mineralization distribution between them were appreciated. For this reason, the analysis was performed with reference to the distribution of pixel frequencies in the 0–255 range of 8-bits.

Three parameters were extracted for each detected peak using the PeakFit software (v. 4.12, Systat Software, San Jose, CA, USA): the mean relative mass density (BMD^r^—mean), the full width at half maxima of the distribution (BMD^r^—fwhm), and the peak area percentage (BMD^r^—area %).

### 2.5. Morpho-Chemical Characterization of the Patient’s Biopsy

The morpho-chemical characterization of the patient’s biopsy was performed combining histological analysis with Fourier Transform Infrared Imaging (FTIRI) spectroscopy. The analyses were carried out at the Advanced Research Instrumentation Laboratory, Department of Life and Environmental Sciences, Università Politecnica delle Marche (Ancona, Italia). Thin adjacent sections (~5 µm thickness) were cut by the FFPE sample and deposited alternatively onto glass slides for the histological evaluation and CaF_2_ optical windows (38 mm × 26 mm × 1 mm size) for the vibrational analysis. All sections were left to air dry for 20 min.

#### 2.5.1. Histology

Histological sections were stained overnight at room temperature using a solution of Alizarin red S (Sigma-Aldrick, Milan, Italy) and Alcian blue (Sigma-Aldrick, Milan, Italy) in a 1:100 ratio according to a modified protocol described by Walker and Kimmel [33]. The two staining solutions were prepared as follows. Considering Alizarin red S, 20 mg of powder was dissolved in 2 mL of 0.5% KOH. Regarding Alcian blue, a stock solution was produced dissolving 20 mg of powder in 5 mL of 70% ethanol. Then, 50 µL of the Alcian blue stock solution was diluted in a solution of 70% ethanol containing 0.0122 g of MgCl_2_ at a final volume of 1 mL. After staining overnight, the sections were then washed in distilled water for 1 min and dehydrated in the ascendant alcohol series (70%, 80%, 95% and 100%; 1 min per step), and cleared in Xylene for 10 min; then, a coverslip was mounted by using the Eukitt reagent (Bio Optica, Milan, Italy). The sample was left to dry overnight and then visualized under an optical microscope, the Zeiss Axio Imager A.2 (Zeiss, Oberkochen, Germany). A microscopic overview of the tissue was obtained at 50× magnification, while the details of the sample were acquired with higher magnification at 200× magnification.

#### 2.5.2. Fourier Transform InfraRed Imaging (FTIRI) Analysis

The FTIR analysis was carried out by using an INVENIO-R interferometer, coupled with a Hyperion 3000 Vis-IR microscope (Bruker Optics, Ettlingen, Germany) and equipped with a Focal Plane Array (FPA) detector operating at liquid nitrogen temperature (Bruker Optics, Ettlingen, Germany). The FPA detector is well suitable for the analysis of non-homogeneous biological samples, such as tissues and cells, since it allows for the collection of IR images which provide information at the morpho-chemical level, correlating, in each point of the sample, the morphological features with the macromolecular composition [34,35,36].

The microphotograph of each section was acquired by using a television camera, and the regions of interest (ROIs) were selected based also on information from histological evidence. IR images were acquired in transmission mode in the 4000–900 cm^−1^ range, with a spectral resolution of 4 cm^−1^; every IR image was 164 × 164 µm^2^ size and it was composed by 4096 pixel/spectra, with a spatial resolution of 2.56 × 2.56 µm^2^; each spectrum was the result of 256 scans. The background spectrum of a clean area of the CaF_2_ optical window was always acquired before each IR image acquisition. Raw IR images obtained in this way were then subjected to the Atmospheric Compensation and Vector Normalization routines in the whole spectral range of acquisition, respectively, for discarding the contribution of atmospheric carbon dioxide and water vapor and avoiding artifacts deriving from thickness differences.

IR images were then integrated under specific spectral intervals to generate false color images, which allow to identify, within the mapped areas, the regions characterized by a different biochemical composition: an arbitrary color scale was used, with blue color indicating areas with the lowest absorbance values, while pink/violet those with the highest ones.

The IR spectra representative of these different areas were extracted, cut in the 1800–900 cm^−1^ range, vector-normalized, and two-points baseline linear fitted. The height of specific peaks, representative of the inorganic and organic matrices, was used to calculate definite spectral parameters.

The acquisition of IR images and all the spectral treatments were performed by the software OPUS 7.1 (Bruker Optics, Ettlingen, Germany).

### 2.6. Statistical Analysis

XμCT, PhC-XμCT, and FTIRI data were submitted to statistical analysis. Descriptive statistics were analyzed using the software package Prism 10.4.1 (GraphPad Software, San Diego, CA, USA). Data distributions were checked for normality by the D’Agostino & Pearson, Anderson–Darling, Kolmogorov–Smirnov and Shapiro–Wilk tests, and homogeneity of variances was assessed by the F-test. Multivariate analyses were performed by Spearman’s rank matrices. The correlations were classified as “very weak” (ρ = 0 ÷ 0.20), “weak” (ρ = 0.20 ÷ 0.40), “moderate” (ρ = 0.40 ÷ 0.60), “strong” (ρ = 0.60 ÷ 0.80) and “very strong” (ρ = 0.80 ÷ 1.0). “Strong” and “very strong” correlations with *p* < 0.05 were considered statistically significant. A *p*-value of <0.05 was accepted as statistically significant for all tests. * *p* < 0.05; ** *p* < 0.01; *** *p* < 0.001; **** *p* < 0.0001.

## 3. Results

The proposed study was conducted in two phases: the first phase involved the analysis of nine cubic scaffolds as fabricated; the second phase entailed the examination of a patient’s biopsy specimen obtained from a sinus lift procedure performed using the same biomaterial.

In the initial phase, the workflow involved several steps: first, the as-fabricated β-TCP scaffold was characterized using scanning electron microscopy (SEM) and X-ray diffraction (XRD) to assess its surface morphology and crystalline structure. Subsequently, micro-computed tomography (XμCT) scans were acquired to perform morphometric analysis of the scaffold’s microarchitecture. A compressive load test was then performed until failure to evaluate mechanical performance. Following mechanical testing, XμCT imaging was repeated on the fractured specimen to analyze the failure modes and structural integrity. Finally, the fractured sample was subjected to SEM analysis to investigate fracture surfaces and microstructural features in detail.

In the second phase, the biopsy obtained from the patient was initially subjected to XμCT imaging to acquire morphometric data regarding the mineralized tissue, focusing on the microarchitecture of the trabecular structure. Subsequently, the same sample was imaged using phase-contrast imaging based on synchrotron radiation (PhC-XμCT) to analyze the network of osteocyte lacunae within the newly formed bone in contact with the residual β-TCP graft biomaterial. Finally, the biopsy was sectioned into slices suitable for histological analysis and FTIRI to investigate tissue organization at the macromolecular level.

### 3.1. Crystalline Structure of the β-TCP Scaffold

X-ray diffraction (XRD) patterns of the raw powder used for scaffold production and the scaffold top surface are reported in Figure S1a, along with the Rietveld refinement results, as shown in Figure S1b,c. Patterns are shown in the reduced angular range 2θ = 35–70°, where the most intense peaks of β-TCP are found. The experimental lattice parameters of the β-TCP scaffold and powder estimated from the Rietveld refinement are reported in Figure S1b and Figure S1c, respectively. Such lattice parameters are in close agreement with the nominal values reported in the PDF2-ICDD file for the β-TCP phase.

### 3.2. Morphometric Analysis of the β-TCP Scaffold

#### 3.2.1. Surface Morphology

**Figure 2:**
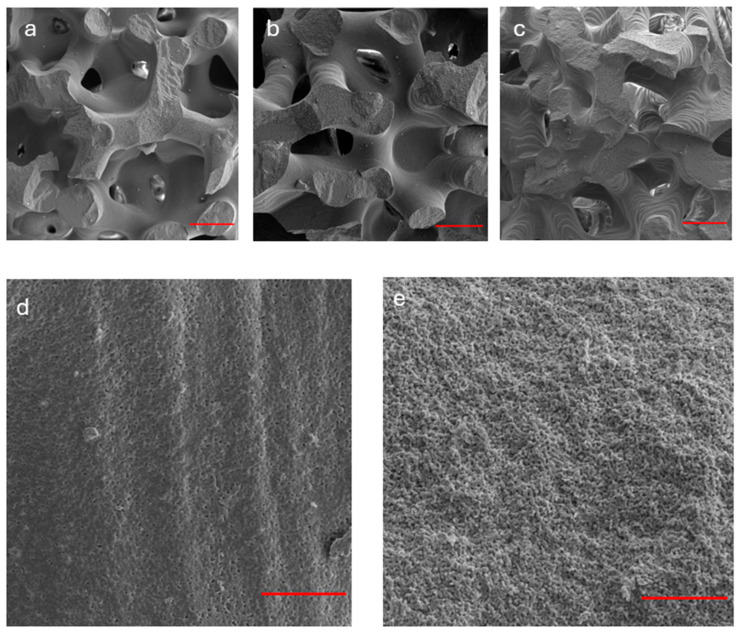
SEM analysis of β-TCP scaffolds (before and after fracture). (**a**–**c**) The observation of the fracture surfaces of three representative samples indicates a predominantly brittle fracture, more pronounced along the branches rather than at the nodes of the trabecular structure. Red bar: 500 µm; (**d**) non-fractured surface: the printing patterns are evident with layer spacing consistent with the nominal value of 25 µm. Red bar: 50 µm; (**e**) fractured surface: an increased microporosity (1–5 µm, in diameter) was observed. Red bar: 50 µm.

SEM images of scaffolds surfaces are reported in Figure 2 in both fractured (Figure 2a–c,e) and not fractured (Figure 2d) conditions. The observation of the fracture surfaces showed that the fracture is primarily brittle, more frequent along the branches of the trabecular structure rather than at the intersections of the nodes. Regarding the unbroken surface, the printing patterns are clearly visible, with a spacing between the layers that is close to the nominal value of 25 µm (Figure 2d). On the fractured surface, instead, an increase in microporosity is observed (Figure 2e), with pores measuring between 1 and 5 µm in diameter, which was also confirmed by EDS analysis, which reported weight percentages of Ca and P not normalized that are slightly lower in the fractured areas than in not fractured (Figure S2a,b). The amounts of Ca and P (in at.%) obtained by EDS analysis performed on the complete set of scaffolds, within experimental uncertainties the Ca/P atomic ratio, are unvaried, suggesting unchanged stoichiometry of β-TCP in all the analyzed samples. It is worth noting that the values of the Ca/P atomic ratio reported in Figure S2a for a representative sample, within the experimental error, agree with the nominal value for stoichiometric β-TCP (Ca/P = 1.5), as obtained from the chemical formula Ca_3_(PO_4_)_2_.

#### 3.2.2. Microarchitecture

**Figure 3:**
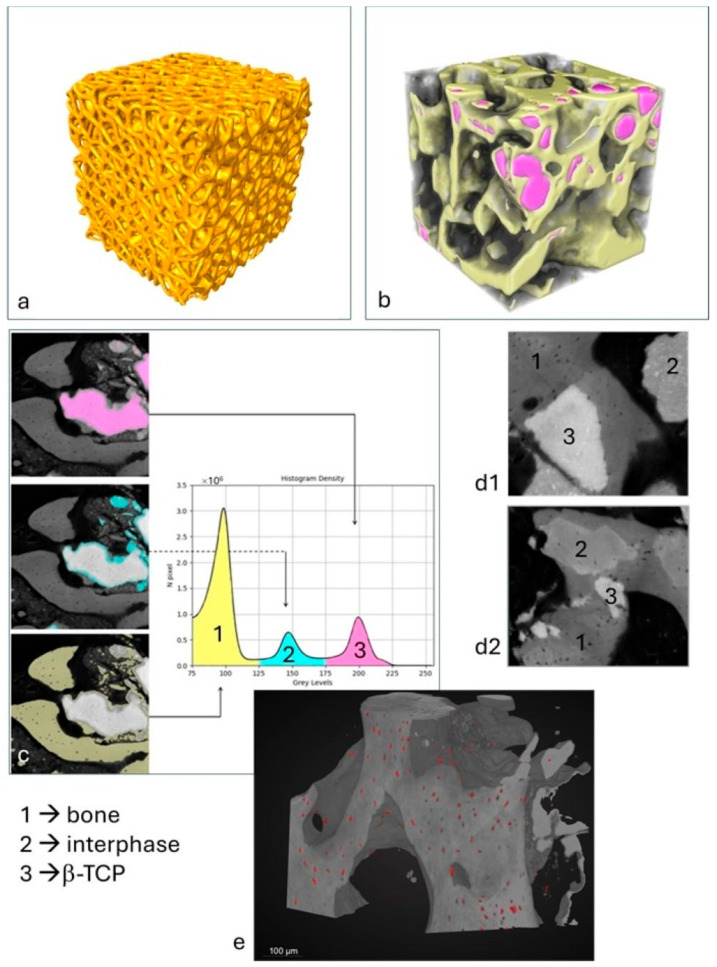
XμCT and PhC-XμCT imaging analysis: (**a**) scaffold microarchitecture in a representative sample; (**b**) 3D XμCT reconstruction of a sub-volume in the patient’s biopsy: yellow: newly formed bone; pink: residual β-TCP; (**c**) histogram of densities in a representative area of the patient’s biopsy: peak 1 = newly formed bone; peak 2 = interphase; peak 3 = residual β-TCP. (**d1**,**d2**) Other representative areas of the patient’s biopsy as imaged by PhC-XμCT with evidence of the biomaterial integration and resorption; (**e**) representative sub-volume of the patient’s biopsy: osteocyte lacunae segmented by the neural net are represented in red.

All nine scaffolds exhibited a microarchitecture, as shown in Figure 3a, that closely resembled the original nominal design. The internal microstructure is illustrated in Supplementary Video S1, which showcases a representative sample from the group. The morphometric parameters of the scaffolds, obtained via XμCT prior to compressive loading, are summarized in Table 1. These parameters were calculated using the BoneJ plugin—an ImageJ (v. 1.53q) extension specialized for bone image analysis [37].

Pre-loading assessments revealed that the scaffolds were homogeneous, with reproducible morphometric metrics and shape complexity, as indicated by low standard deviations (SD). This suggests highly isotropic structures, with an anisotropy DA close to zero. The trabeculae exhibited an average thickness of approximately 400 μm and an average inter-trabecular distance of about 550 μm, with a volume fraction near 50%. Additionally, parameters such as fractal dimension and connectivity demonstrated high stability, further confirming the reproducibility of the manufacturing process.

**Figure 4:**
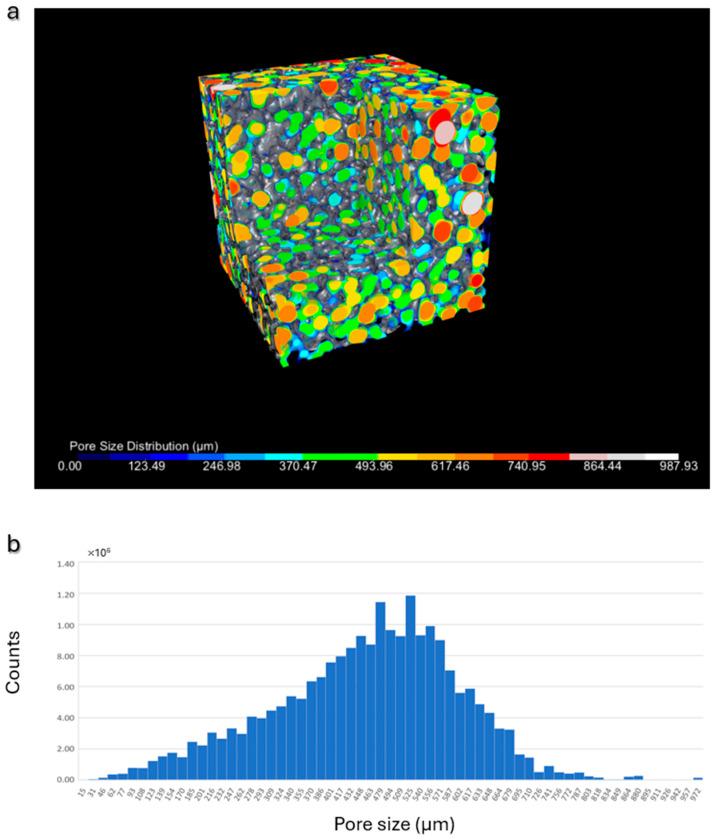
Pore size analysis: (**a**) representative β-TCP scaffold: 3D visualization of the pores, color-coded according to their dimensions; (**b**) histogram illustrating the pore size distribution in a representative scaffold.

Furthermore, considering the significant influence of pore dimensions and distribution on the bioactive properties of the scaffold, an analysis was performed to determine both the spatial pore distribution and the pore size distribution. The results, depicted through color-coding in Figure 4a and the histogram illustrating the pore size distribution in Figure 4b, collectively demonstrate a broad range of pore sizes, with dimensions exceeding 100 µm and an average value of approximately 500 µm, in accordance with the literature recommendations for achieving osteoconductivity.

Post-compressive loading XμCT analyses are detailed in Table S2, which reports parameters including volume percentage, trabecular thickness, fractal dimension, and connectivity after fracture. Notably, the structure exhibited increased anisotropy post-loading, as evidenced by an elevated DA, likely attributable to the applied mechanical stress.

### 3.3. Compressive Load Tests

**Figure 5:**
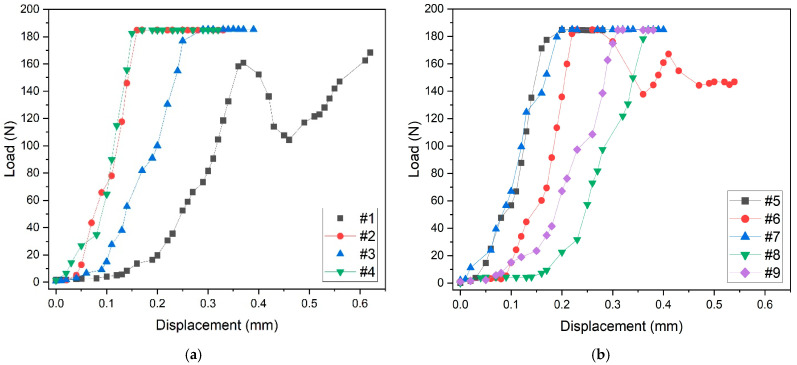
Compressive load tests: (**a**) mechanical behavior of samples tested in the build direction, (**b**) mechanical behavior of samples tested in the random direction.

The mechanical response of scaffolds to compressive tests is shown in Figure 5. Samples tested in the build direction almost show a similar behavior, except for sample #1, which shows oscillations of the curve before fracture (Figure 5a). Such oscillations are typical of scaffolds built layer by layer by additive manufacturing techniques. On compression, the failure of one or two struts of the layer causes the collapse of the entire layer that occurs in correspondence with a minimum of the curve. The collapse of the layer produces a compacted structure responsible for the curve shape after this event. This mechanism is repeated layer by layer, causing the oscillating shape of the curve. As the collapse of the layers occurs from the outside to the inside during compression, the general shape of the first part of the curve is not perturbed. However, the slope of this part of the curve up to about 170 N load may differ from sample to sample, due to small differences in the positioning of the sample on the compression stage.

The mechanical behavior of samples tested in random direction is shown in Figure 5b.

Also, in this case, the general shape of the curves is similar, except for sample #6, which was probably tested in the build direction because of the oscillations of the curve. The maximum applied load for all samples is about 180 N, and the maximum displacement before fracture is about 0.4 mm. It is worth noting that the two oscillating curves of samples #1 (Figure 5a) and #6 (Figure 5b) reach the maximum value of about 0.6 mm displacement before fracture.

### 3.4. Spearman Correlative Analysis

The Spearman correlation analysis (Supplementary Figure S3a), examining the relationships between scaffold morphometric parameters and mechanical loading indices across the nine investigated scaffolds, identified the following significant correlations: (a) trabecular thickness (Tb.Th) exhibits a strong positive correlation with Young’s modulus (ρ = +0.70, *p* = 0.043); (b) maximum trabecular thickness (Tb.Th.Max) shows a strong positive correlation with maximum load capacity (ρ = +0.78, *p* = 0.017); (c) the anisotropy degree (DA) is strongly positively correlated with specific energy absorption (ρ = +0.68, *p* = 0.050).

### 3.5. The Patient’s Biopsy

#### 3.5.1. Histological Analysis

**Figure 6:**
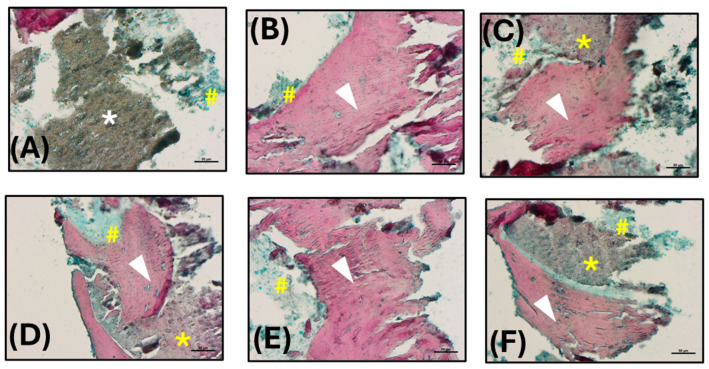
Histological analysis: (**A**–**F**) Histological images obtained by Alcian blue and Alizarin red S staining (200× magnification; scale bars 50 µm). Hydroxyapatite deposition is red stained (white arrow heads), while the collagen component is blue (yellow hashes). The β-TCP appears not stained, in grey (white asterisk), or lightly stained in red in the region of new bone formation (yellow asterisks).

The analysis of the histological images obtained by Alcian blue and Alizarin red S staining is described below. A grey-colored region lacking staining and attributable to the β-TCP scaffold was observed (white asterisk; Figure 6A), while the bone tissue was well evident in red, owing to the binding with hydroxyapatite (white arrow heads, Figure 6B–F). Some small light blue colored areas were also found, probably associated with collagen deposition (yellow hashes, Figure 6A–F). Interestingly, it was possible to detect some regions light red stained (yellow asterisks, Figure 6C,D,F) suggesting the beginning of the mineralization process and hence the formation of new bone.

#### 3.5.2. Microarchitecture of the Trabecular Structure

The bioptic specimen analyzed via bench-top XμCT (Figure 3b) exhibited a microarchitecture characterized by the following parameters: the specific volume percentage (Vol.%)—representing the combined volume of newly formed bone and residual mineralized β-TCP scaffold—was approximately 55.4%. The mean trabecular thickness (Tb.Th) was 195 ± 74 μm, with a maximum value of 428 μm, while the mean trabecular spacing (Tb.Sp) was 194 ± 88 μm, reaching up to 379 μm. Additionally, the connectivity density (Conn.D; mm^−3^) was measured at 104, the degree of anisotropy (DA) was 0.327, and the fractal dimension (FD) was calculated to be 2.44.

Furthermore, the examined biopsy sample, analyzed by PhC-XμCT with synchrotron radiation, shows three distinct absorption peaks, each corresponding to phases with different densities (Figure 3c,d). The first peak is attributed to mature bone tissue and corresponds to the phase with the lowest density (Phase 1, in Figure 3c,d). The second peak represents an interphase, which will probably disappear for regenerative periods much longer than six months after grafting (Phase 2, in Figure 3c,d). The third and densest peak indicates residual β-tricalcium phosphate (β-TCP) (Phase 3, in Figure 3c,d). The volumetric percentage of the newly formed bone was measured to be approximately 28.4 (±14.2) vol%, and the interphase was around 15.4 (±11.4) vol%, while the residual biomaterial was found to be approximately 11.6 (±10.4) vol%. The large standard deviations for the three parameters suggest a non-homogeneity of regeneration within the different biopsy areas. This confirms the fact that the regenerative process is not yet complete after 6 months of biomaterial grafting. The Spearman correlation analysis investigating the relationships among bone, interphase, and biomaterial indices across the nine regions analyzed in the biopsy (Supplementary Figure S3b) revealed the following significant correlations: (a) the bone peak volume percentage (bone-vol%) shows a strong negative correlation with the interphase peak volume percentage (interphase-vol%) (ρ = −0.73, *p* = 0.031); (b) the interphase peak mean density (interphase-mean) exhibits a strong negative correlation with the interphase peak volume percentage (interphase-vol%) (ρ = −0.80, *p* = 0.013).

#### 3.5.3. Osteocyte Lacunae Network in the Patient’s Biopsy

Figure 3e shows, in a representative sub volume, the distribution of osteocyte lacunae; most of the lacunae have a size ranging from 400 to 500 µm^3^, variously distributed among the different areas of the sample, in the bone phase portion.

The lacunar SCFs that were evaluated by the synchrotron analysis are reported in Table 2, introducing mean, standard deviation and 95% confidence interval as descriptive parameters.

Spearman’s correlation analysis examining the relationships between the shape complexity characteristics of osteocyte lacunae (Supplementary Figure S3c) in the nine ROIs of the examined biopsy identified a significant positive correlation between LacV and LacV/S (ρ = +0.90, *p* = 0.005), confirming that larger lacunae correspond to more spherical shapes, i.e., presumably younger lacunae.

#### 3.5.4. Macromolecular Analysis

For better comprehension of the hyperspectral imaging analysis that follows, the IR spectra of pure β-TCP and bone hydroxyapatite (HA) were collected (Supplementary Figure S4). As expected, β-TCP shows different spectral features respect to a mature bone. In fact, in the IR spectrum of pure β-TCP, only the bands associated with the vibrations of phosphate groups are present (~1117 cm^−1^, ~1018 cm^−1^, ~970 cm^−1^, and ~944 cm^−1^) [38]. Conversely, the IR spectrum of a bone sample is characterized by the Amide I (~1656 cm^−1^), II (~1551 cm^−1^), and III (~1282 cm^−1^, ~1239 cm^−1^, and ~1203 cm^−1^) bands of the organic matrix (proteins, and collagen) and by the peaks of phosphates (~1100 cm^−1^, and ~1032 cm^−1^, attributable, respectively, to poor and well-crystallized stoichiometric hydroxyapatite, and ~960 cm^−1^) and carbonates (~1451 cm^−1^, and ~1415 cm^−1^) of the inorganic component [39].

**Figure 7:**
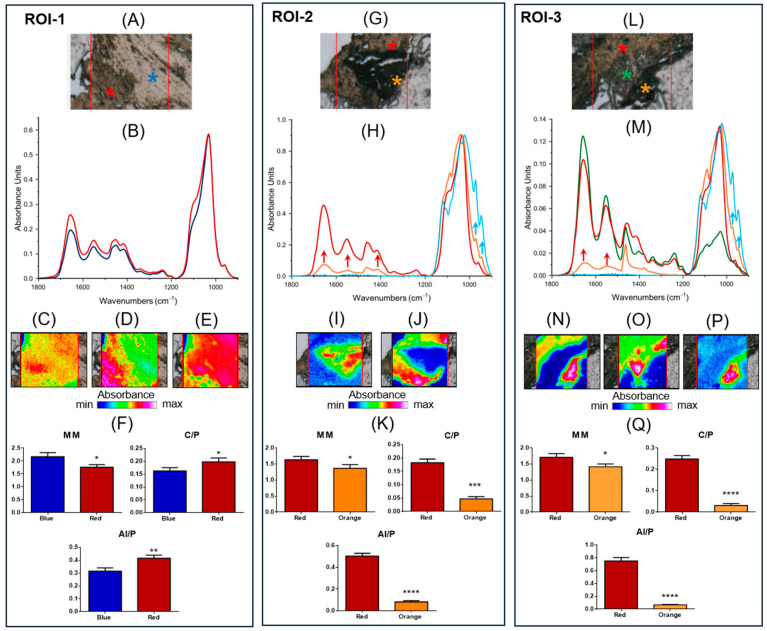
FTIR hyperspectral Imaging analysis: ROI-1: (**A**) Microphotograph of ROI-1 (red box): red and blue asterisks indicate the presence of areas with different macromolecular composition. (**B**) Absorbance IR spectra representative of the two areas (red and blue lines). False color images obtained integrating IR images under the bands at (**C**) ~1412 cm^−1^. (**D**) ~1100 cm^−1^, and (**E**) ~1032 cm^−1^. (**F**) Spectral parameters: Mineral Maturity Index (MM), Carbonates-to-Phosphates ratio (C/P), and Mineral-to-Matrix ratio (AI/P). ROI-2: (**G**) Microphotograph of ROI-2 (red box): red and orange asterisks indicate the presence of two regions with different macromolecular composition. (**H**) Absorbance IR spectra representative of the two areas (red and orange lines) and of pure β-TCP (light blue line). False color images obtained integrating under the bands at (**I**) ~970 cm^−1^, and (**J**) ~1032 cm^−1^. (**K**) Spectral parameters: Mineral Maturity Index (MM), Carbonates-to-Phosphates ratio (C/P), and Mineral-to-Matrix ratio (AI/P). ROI-3: (**L**) Microphotograph of ROI-3 (red box): red, green, and orange asterisks indicate the presence of areas with different macromolecular composition. (**M**) Absorbance IR spectra representative of the three areas (red, green, and orange lines) and of pure β-TCP (light blue line). False color images obtained integrating under the bands at (**N**) ~1032 cm^−1^, (**O**) ~1656 cm^−1^, and (**P**) ~970 cm^−1^. (**Q**) Spectral parameters: Mineral Maturity Index (MM), Carbonates-to-Phosphates ratio (C/P), and Mineral-to-Matrix ratio (AI/P). False color images were built by using an arbitrary color scale, blue color indicating areas with the lowest absorbance values (min absorbance), while pink/violet those with the highest ones (max absorbance). As regards the statistical analysis, data are shown as mean ± SD; asterisks over histograms indicate statistically significant differences (* *p* < 0.05; ** *p* < 0.01; *** *p* < 0.001, and **** *p* < 0.0001).

The hyperspectral imaging analysis that follows was performed on specific regions of interest (hereinafter named ROI-#) identified on the sections cut from patient’s biopsy, as well as on the base of the histological evidence. In ROI-1, the presence of two different tissue areas stands out clearly (Figure 7A). Both regions are mainly composed of hydroxyapatite, as suggested by the spectral profiles in Figure 7B. This is also confirmed by false color images calculated on the bands at ~1412 cm^−1^, attributable to inorganic carbonates (Figure 7C), ~1100 cm^−1^ (Figure 7D), and ~1032 cm^−1^ (Figure 7E); the latter two were assigned to phosphate groups, respectively, in poor and well-crystallized stoichiometric HA. The orange/red color in Figure 7C suggests a homogeneous distribution of carbonate groups and confirms the presence of bone tissue in the whole analyzed area. Nevertheless, the different colorations in Figure 7D,E uphold a distinct degree of crystallization: the lower left part of Figure 7D (red/pink colored), corresponding to the red asterisk region, is characterized by the presence of poor HA, while the upper right part of Figure 7E (red/pink colored), corresponding to the blue asterisk area, is composed of well-crystallized stoichiometric HA. Finally, to ascertain the quality of the bone tissue, the following spectral parameters were analyzed [39,40]: (i) the Mineral Maturity index (MM), calculated as ratio between the intensities of the peaks at ~1032 cm^−1^ and ~1100 cm^−1^, which is diagnostic for the level of bone crystallinity; (ii) the Carbonates-to-Phosphates ratio (C/P), obtained by ratioing the intensities of the peaks at ~1415 cm^−1^ and ~1032 cm^−1^, which indicates the level of substitution of carbonates in the apatite lattice; (iii) the Mineral-to-Matrix ratio (AI/P), calculated as ratio between the intensities of the peaks at ~1656 cm^−1^ and ~1032 cm^−1^, which signifies the level of mineralization of the bone and can be related to its mechanical performance. In the region identified by the blue asterisk, the MM was higher (*p* < 0.05), while the C/P and AI/P were lower (respectively, *p* < 0.05 and *p* < 0.01), indicating, in this area, a higher level of bone crystallinity and a lower amount of carbonate groups in the lattice apatite (Figure 7F).

ROI-2 is also characterized by two different areas (red and orange asterisks in Figure 7G). In Figure 7H, the IR spectra representative of the two regions are shown together with that of pure β-TCP (light blue line). The analysis of the spectral profiles suggests, in the red asterisk region, the presence of bone tissue with poorly crystallized HA. Conversely, the IR spectrum of the orange asterisk region is characterized not only by the bands typical of a bone (such as the bands at ~1656 cm^−1^ and ~1551 cm^−1^, named AI and AII, and associated with the organic matrix, and ~1412 cm^−1^ attributable to the inorganic carbonates in the apatite lattice; red arrows), but also by the peaks at ~970 cm^−1^, and ~943 cm^−1^, related to β-TCP (light blue arrows). This was also confirmed by false color images obtained integrating IR images, respectively, under the bands at ~970 cm^−1^ (Figure 7I) and ~1032 cm^−1^ (Figure 7J). All these findings allow us to hypothesize, in the orange asterisk region, the beginning of the mineralization process induced by β-TCP, as also suggested by the analysis of the spectral parameters (Figure 7K); in fact, in the orange asterisk region, the MM, C/P and AI/P were lower (respectively, *p* < 0.05, *p* < 0.001, and *p* < 0.0001) with respect to those calculated in the red asterisk one, confirming, in this area, a new immature bone with low crystallinity and a low amount of carbonates and organic matrix.

The third investigated region, named ROI-3, appears more complex due to the presence of various biochemical components (Figure 7L). The analysis of the spectral profiles (Figure 7M) indicates, in the red asterisk region, the presence of bone tissue, with poorly crystallized HA; conversely, the orange asterisk region shows both the peaks associated with the organic matrix (such as AI and AII; red arrows), and those related to β-TCP at ~970 cm^−1^ and ~943 cm^−1^ (light blue arrows), indicating the presence of new bone formation. Interestingly, an additional region at the interface of those already described was evidenced (green asterisk) characterized as extracellular matrix, as suggested by the high intensities of Amide I, II and III bands, and by the low intensity of a convoluted band attributable to phosphates. The same results were evidenced by false color images integrated under the bands at ~1032 cm^−1^ (Figure 7N), ~1656 cm^−1^ (Figure 7O), and ~970 cm^−1^ (Figure 7P). As expected, the spectral parameters MM, C/P, and AI/P showed lower values in the orange asterisk region, confirming, in this area, the presence of new bone formation with scarce crystallinity and a low amount of carbonates and organic components (Figure 7Q).

## 4. Discussion

The body of literature on bone substitute biomaterials (BSBs) proposed for jawbone regeneration is vast, reflecting an ongoing pursuit to identify ideal scaffolds capable of effectively substituting autologous bone. This need is particularly pronounced in sinus lift elevation procedures, which are performed when an insufficient bone volume is available for the placement of dental implants. Outcomes associated with these BSBs exhibit considerable variability, influenced not only by individual patient characteristics but also by specific initial surgical conditions, the chemical composition of the biomaterial, and its resorption kinetics and profile (as outlined in Table S3). While some biomaterials, endowed with favorable biocompatibility and biomechanical properties, promote early healing compatible with immediate implant loading, they often exhibit minimal or even negligible resorption over time, potentially leading to several complications, including infections or granuloma formation, if the residual biomaterial is not fully integrated or if unassimilated fragments are present [41,42]. Moreover, impaired implant osseointegration may occur if the biomaterial is not fully absorbed or integrated into the bone, resulting in inadequate anchorage of the dental implant and an increased risk of implant failure [43]. Conversely, other materials favor rapid resorption to facilitate native bone regeneration but may lack adequate mechanical stability during the critical early phases of healing, thus precluding immediate functional loading [44]. The ongoing challenge, therefore, remains the development of a biomaterial that simultaneously balances biological integration, mechanical stability, and controlled resorption, addressing the multifaceted demands of jawbone regeneration.

This study highlights the potential of lithography-based manufactured β-tricalcium phosphate (β-TCP) scaffolds as viable biomaterials for sinus lift elevation, particularly in complex cases involving severe maxillary atrophy. The integration of advanced additive manufacturing techniques, detailed structural characterization, and in vivo outcomes provides a comprehensive understanding of scaffold performance and biological tissue response.

From a material perspective, crystalline analysis through X-ray diffraction (XRD) confirmed the exclusive presence of the β-TCP phase, with no significant deviations in stoichiometry. Scanning electron microscopy observations revealed the presence of a uniform microporous lattice, with pores of average diameters of a few micrometers. Moreover, after compressive loading, it revealed the presence of brittle fracture, primarily along trabecular branches but almost excluding the trabecular nodes. The microarchitectural analysis demonstrated that the additive manufacturing process reliably reproduced the intended porosity and trabecular features, without significant deviations between one sample and another, with average macro/micro porosity conducive to vascularization and biological infiltration. In follow-up studies, the Brunauer–Emmett–Teller (BET) theory [45] will be employed for surface area analysis. This will enable a more precise characterization of the scaffold’s surface properties, thereby enhancing our understanding of its interactions with the biological environment.

The mechanical performance of biomaterials used for sinus augmentation is critically important to the success of this surgical procedure. Numerous studies have evaluated the effectiveness of various grafting materials, revealing significant differences in outcomes related to bone regeneration and postoperative complications. While the maximum compressive load supported by these scaffolds is slightly lower than the values measured on lithography-based manufactured biphasic calcium phosphate scaffolds and comparable to that of those that are traditionally sintered, it has also been shown that the complete reabsorption of β-TCP, combined with a more suitable microarchitecture of the regenerated tissue, makes this scaffold extremely promising for maxillary bone engineering [10,46].

Moreover, post-mechanical compressive testing indicated the preservation of macrostructural features, although increased anisotropy suggested some structural reorganization under load, which is consistent with the correlation between microstructural parameters and mechanical resilience observed in Spearman’s analysis. By Spearman’s analysis, Young’s modulus was observed to be strongly positively correlated to trabecular thickness; this occurs because thicker trabeculae provide greater resistance to compression and applied mechanical forces, enhancing the scaffold’s ability to withstand loads without deforming. This greatly mimics the natural trabecular microarchitecture of bone that is designed to efficiently distribute forces: as trabecular thickness increases, the bone becomes less susceptible to microfractures and other deformations that would reduce its strength. This results in greater strength and, consequently, a higher Young’s modulus. Moreover, the anisotropy degree of the scaffold was found to strongly positively correlate with specific energy absorption; indeed, anisotropic scaffolds can be designed to better withstand specific mechanical loads, such as compression, by aligning the structure in ways that enhance energy absorption. This helps to optimize the scaffold’s performance, particularly in dynamic or high-stress environments, like in peri-implant bone, by enabling it to absorb and dissipate energy more effectively.

Biologically, the in vivo evaluation corroborated the biocompatibility and osteoconductive of the β-TCP scaffold, as evidenced by histological analysis and combined XμCT and synchrotron imaging. The detection of nascent mineralized bone tissue, indicated by histological staining, suggests the initiation of osteogenesis within the scaffold, while XμCT and PhC-XμCT analyses revealed the ongoing resorption of residual β-TCP concurrent with new bone formation. Volumetric quantification demonstrated a complex regenerative landscape, with substantial heterogeneity in both mineralization stages and tissue integration, reflecting the typical temporal dynamics of bone healing and scaffold resorption while maintaining overall functional microarchitecture. The osteocyte lacunae analysis via synchrotron phase-contrast XμCT and AI segmentation revealed that the osteocyte network exhibits morphological features consistent with ongoing bone maturation, with lacunae sizes and shapes suggesting variable stages of osteoblast-derived new bone. The positive correlation between lacunar volume and sphericity underscores the relationship between osteocyte morphology and bone vitality, offering potential biomarkers for tissue regenerative quality.

Furthermore, morpho-chemical characterization via Fourier Transform Infrared Imaging (FTIRI) depicted a landscape of mineralization maturity, with spectral parameters indicating heterogeneous levels of bone matrix organization and mineral crystallinity. Regions with higher mineral maturity indices and well-consolidated crystalline domains highlight the progressive ossification and maturation trajectory following grafting, aligning with the objective of achieving functional loading through dental implants.

Overall, this integrated approach, combining scaffold design and biomechanical characterization, in vivo biological response, and advanced imaging, underscores the potential of lithography-based manufactured β-TCP scaffolds in maxillofacial regenerative procedures, particularly sinus lift augmentation. Future research should focus on optimizing scaffold production to furtherly standardize microarchitectural parameters and, consequently, mechanical performance, extending in vivo observation periods to monitor long-term remodeling.

In any case, the outcomes of this study contribute to the validation of the proposed scaffold. This constitutes a necessary, yet distinct and preliminary phase, which precedes the evaluation of clinical applicability—a process that, by definition, entails the establishment of specific inclusion and exclusion criteria for participant selection, the definition of intervention (test) and control groups, and the implementation of procedures such as randomization, blinding, and the identification of predefined endpoints.

## 5. Conclusions

This study rigorously investigated the synergistic advantages of combining LCM production processes with β-TCP material for bone grafts, focusing on their clinical applicability in maxillofacial regenerative procedures, specifically sinus lift augmentation. Through a multidisciplinary approach, this research quantified key properties of the resulting β-TCP scaffolds, revealing several critical findings:

Microarchitectural uniformity and tissue integration: The scaffolds demonstrated uniform porosity and reliably reproduced trabecular-like structures. This architecture is highly advantageous for promoting vascularization and facilitating tissue ingrowth, crucial aspects for successful bone regeneration.

Mechanical performance: While it exhibited slightly lower compressive strength compared to biphasic calcium phosphate alternatives, the scaffold’s mechanical properties were deemed sufficient for clinical application. Furthermore, positive correlations were observed between trabecular thickness and Young’s modulus, and between anisotropy and specific energy absorption. These correlations suggest optimized mechanical resilience under physiological loads, indicating the scaffold’s capacity to withstand the forces encountered in the maxillofacial region.

Biocompatibility and osteoconductivity: This study confirmed the scaffold’s biocompatibility and osteoconductivity, evidenced by active osteogenesis and concurrent scaffold resorption. This indicates the material’s ability to integrate with host tissue and promote new bone formation while gradually degrading.

Regenerative dynamics and bone maturation: Heterogeneous mineralization and tissue integration patterns were observed, reflecting typical regenerative dynamics. The scaffold maintained its functional architecture throughout this process. Importantly, lacunar analysis indicated ongoing bone maturation, with lacunar morphology identified as a potential marker for assessing regenerative quality.

Future research should prioritize optimizing scaffold production to further standardize microarchitectural parameters and enhance mechanical performance. Additionally, extending in vivo observation periods will be crucial for monitoring long-term remodeling processes, and exploring functional outcomes in broader patient populations will provide a more comprehensive understanding of clinical efficacy.

## Figures and Tables

**Table 1:** β-TCP scaffold (as produced) microarchitectural parameters—descriptive statistics.

β-TCP Scaffold (as Produced)	Mean	Std.Dev.	95% CI of Mean
BV/TV (%)	48	4	45 to 51
Porosity (%)	52	4	49 to 55
Tb.Th Mean (µm)	387	16	375 to 399
Tb.Th Std Dev (µm)	97	10	89 to 104
Tb.Th Max (µm)	702	60	656 to 748
Tb.Sp Mean (µm)	546	93	475 to 618
Tb.Sp Std Dev (µm)	330	137	225 to 435
Tb.Sp Max (µm)	2377	624	1897 to 2856
DA	0.195	0.015	0.183 to 0.207
Fractal Dimension	2.52	0.08	2.46 to 2.59
Conn. D (mm^−3^)	7	2	6 to 9

**Table 2:** Osteocyte lacunae network shape complexity features (SCF) in the patient’s biopsy—descriptive statistics.

Patient’s Biopsy—SCFs	Mean	Std.Dev.	95% CI of Mean
LacVol (µm^3^)	426	42	391 to 461
LacV/S (µm)	1.24	0.05	1.20 to 1.28
LacAR	0.334	0.019	0.318 to 0.350
LacS	0.825	0.013	0.815 to 0.836
LacNr (mm^−3^) × 10^4^	1.82	0.32	1.55 to 2.09

## Data Availability

The original contributions presented in this study are included in this article and in the Supplementary Materials; further inquiries can be directed to the corresponding authors.

## Supplementary Materials

The following supporting information can be downloaded at: https://www.mdpi.com/article/10.3390/jfb16070237/s1, Table S1: Convolutional neural network. Used training parameters to maximize the performance; Figure S1: X-ray diffraction spectrum of the β-TCP powder and scaffold; Figure S2: EDS data analysis of β-TCP scaffolds; Video S1: 3D reconstruction of a representative β-TCP scaffold; Table S2: β-TCP scaffold (after compressive loading) microarchitectural parameters—descriptive statistics; Figure S3: Spearman’s correlative matrixes. (a) β-TCP scaffold (as produced): microarchitectural and compressive loading parameters; (b) retrieved biopsy (after 6 months of grafting): microarchitectural and bone mineral density parameters; (c) retrieved biopsy (after 6 months of grafting): osteocyte lacunae network shape complexity features; Figure S4: Absorbance IR spectra of β-TCP (light blue line), and bone hydroxyapatite (red line). The spectra are displayed in the 1800–900 cm^−1^ spectral range; the position (in terms of wavenumbers, cm^−1^) of the most significant peaks are indicated at the top; Table S3: Biomaterials performance in sinus lift augmentation—microarchitecture in clinical cases.

## Abbreviations

The following abbreviations are used in this manuscript:

β-TCP	Beta Tricalcium Phosphate
LCM	Lithography-based ceramic manufacturing
RP	Rapid Prototyping
CBCT	Cone-beam computed tomography
CAD	Computer-aided design
XRD	X-ray diffraction
ICDD	International Centre for Diffraction Data
SEM	Scanning electron microscopy
XμCT	X-ray micro-computed tomography
MTS1	Material Testing Stage
PhC-XμCT	Phase-contrast X-ray micro-computed tomography
STP	Syrmep Tomo Project
TIE	Transport of Intensity Equation
SCF	Shape complexity features
ROI	Regions Of Interest
I	infraRed
FTIRI	Fourier Transform InfraRed Imaging
BV/TV	Ratio between mineralized volume and total scaffold volume
Tb.Th	Mean trabecular thickness
Tb.Sp	Mean trabecular spacing
Conn.D	Connectivity Density
DA	Anisotropy Degree
FD	Fractal Dimension
LacV	Mean Lacuna Volume
LacV/S	Mean Lacunar Volume/Surface ratio
LacAR	3D Lacunar Aspect Ratio
LacS	Lacunar Sphericity
LacNr	Lacunar density
BMD^r^	Relative mass density
