# Supplementary material for: Morphometric, Biomechanical and Macromolecular Performances of β-TCP Macro/Micro-Porous Lattice Scaffolds Fabricated via Lithography-Based Ceramic Manufacturing for Jawbone Engineering
Source: J Funct Biomater. 2025 Jun 28;16(7):237. doi: 10.3390/jfb16070237 (PMC12295332; doi:10.3390/jfb16070237)
Supplement: Supplementary file 1 [file jfb-16-00237-s001.zip › jfb-3690191-supplementary.pdf]

Supplementary Material

Table S1. Convolutional Neural Network. Used training parameters to maximize the performance.

U-Net Training Parameters	
Class count	3
Depth level	5
Initial filter count	64
Patch size	32
Stride ratio	0.5
Batch size	32
Epochs number	40
Loss function	Categorical Cross-Entropy
Optimization algorithm	Adadelta
Metrics	Categorical Accuracy
Early stopping	NO
Reduce learning rate on plateau	YES
Training/Validation	90/10

Figure S1. X-Ray Diffraction (XRD) patterns of the β-TCP powder and scaffold. (a) Comparison between the XRD patterns of powder and scaffold. Patterns are reported in square root intensity scale ($[\text{Intensity}]^{1/2}$) to enhance the low diffraction peaks and vertically translated to easy comparison. (b) XRD pattern of the scaffold (red symbol), Rietveld refinement (black line) and curve of residues (magenta line). (c) XRD pattern of the raw powder used to produce scaffolds (red symbol), Rietveld refinement (black line) and curve of residues (magenta line). Position of diffraction peaks for the β-TCP phase of the $Ca_3(PO_4)_2$ compound are shown as vertical lines in (b) and (c).

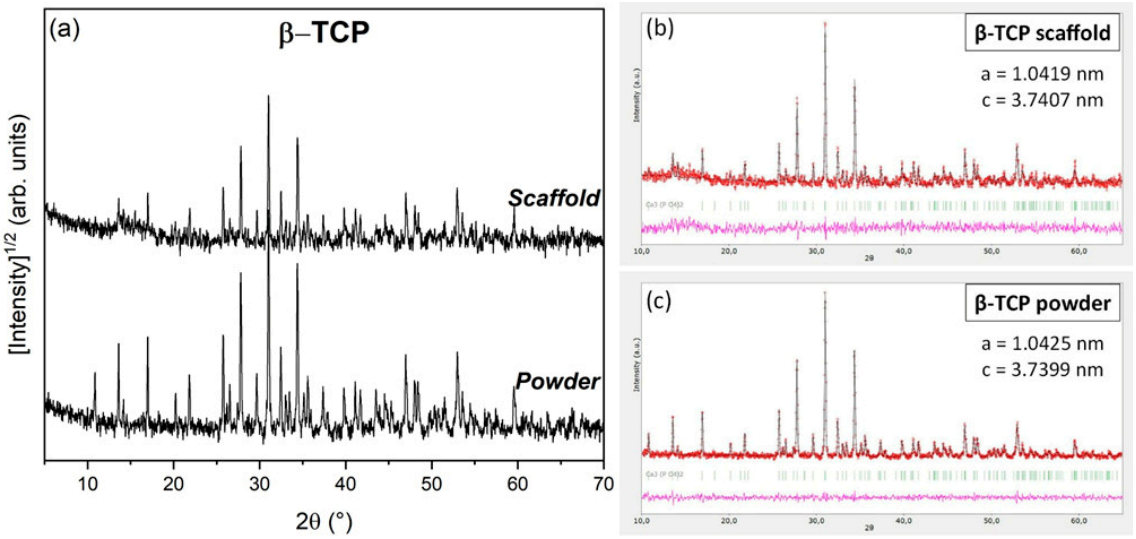

Figure S2. EDS data analysis of β-TCP scaffolds: (a) not-fractured and (b) fractured surfaces; the measured unnormalized wt.% of Ca and P slightly decrease in fractured regions.

a

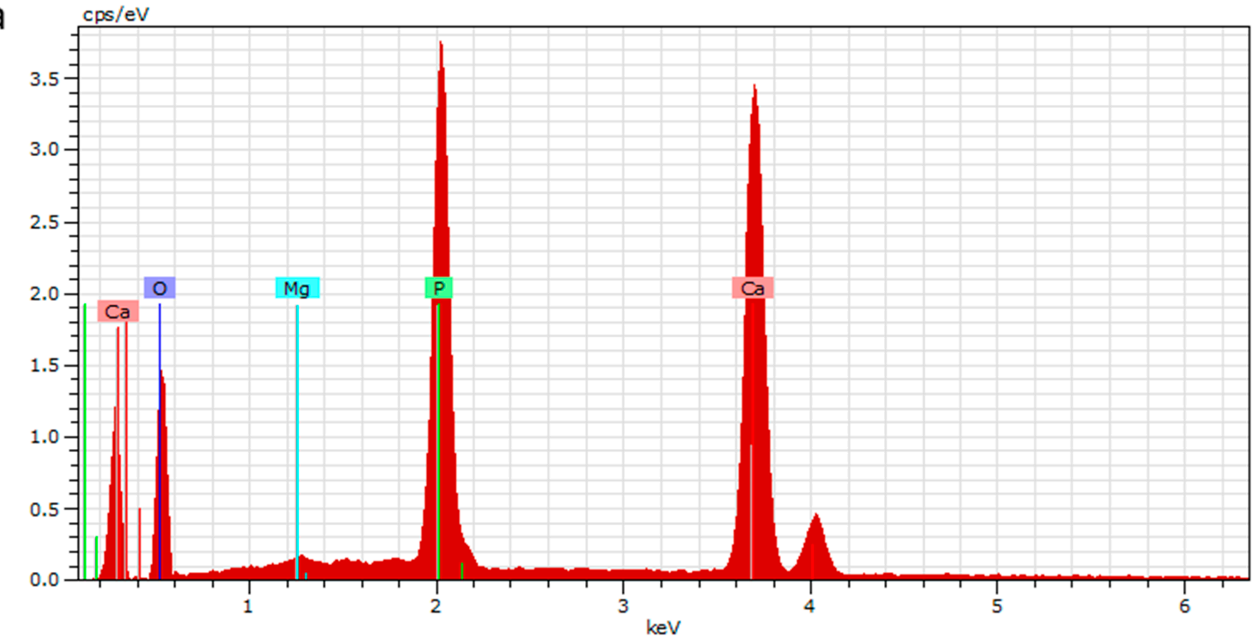

Not Fractured Area: Representative Spectrum Acquisition

Element	Series	unn. C [wt.%]	norm. C [wt.%]	Atom. C [at.%]	Error (1 Sigma) [wt.%]
Calcium	K-series	42.90	42.09	25.94	1.34
Phosphorus	K-series	20.68	20.29	16.17	0.83
Oxygen	K-series	37.99	37.27	57.53	5.87
Magnesium	K-series	0.36	0.35	0.36	0.06
	Total:	101.92	100.00	100.00	

b

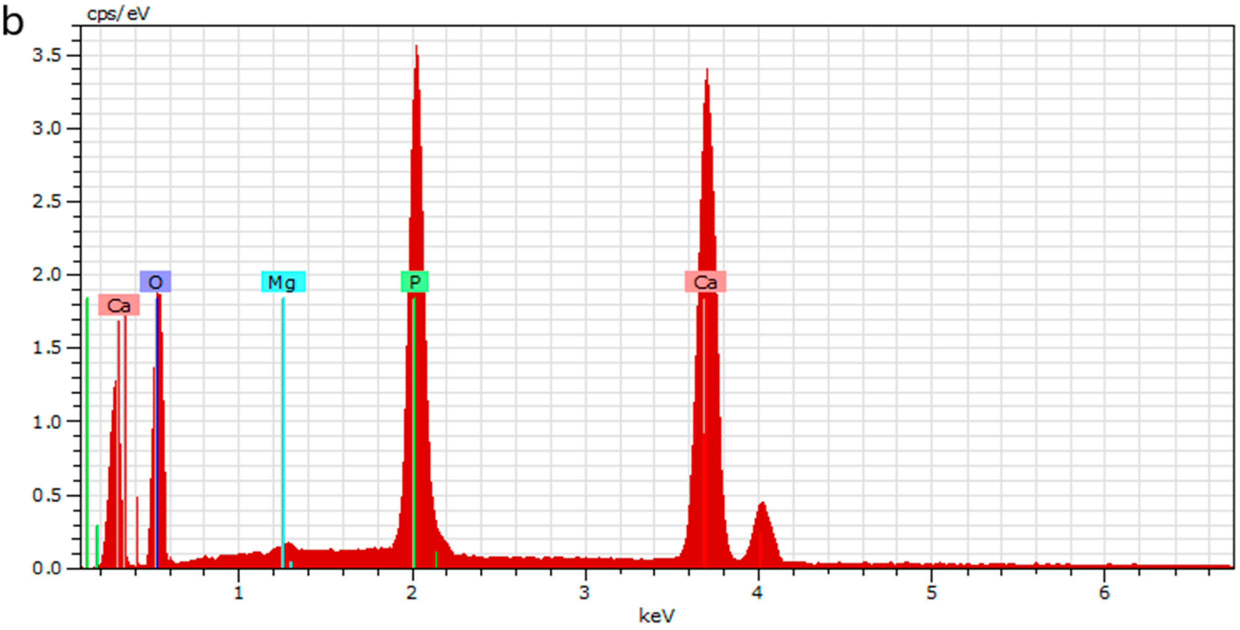

Fractured Area: Representative Spectrum Acquisition

Element	Series	unn. C [wt.%]	norm. C [wt.%]	Atom. C [at.%]	Error (1 Sigma) [wt.%]
Calcium	K-series	39.92	40.02	23.58	1.25
Phosphorus	K-series	16.72	16.76	12.78	0.68
Oxygen	K-series	42.81	42.91	63.34	6.31
Magnesium	K-series	0.32	0.32	0.31	0.05
	Total:	99.76	100.00	100.00	

Video S1 (caption). 3D reconstruction of a representative β-TCP scaffold.

Table S2. β-TCP scaffold (after compressive loading) microarchitectural parameters - Descriptive Statistics.

β-TCP scaffold (as produced)	MEAN	STD.DEV	95% CI
BV/TV (%)	22	2	17 to 28
Tb.Th Mean (µm)	340	10	315 to 365
Tb.Th Std Dev (µm)	107	9	84 to 130
Tb.Th Max (µm)	638	18	592 to 681
DA	0.240	0.067	0.074 to 0.406
Fractal dimension	2.47	0.01	2.45 to 2.48
Conn. D (mm^{-3})	6	1	3 to 9

Figure S3. Spearman correlative matrixes. (a)β-TCP scaffold (as produced): microarchitectural and compressive loading parameters; (b) retrieved biopsy (after 6-months grafting): microarchitectural and bone mineral density parameters; (c) retrieved biopsy (after 6-months grafting): osteocyte lacunae network shape complexity features.

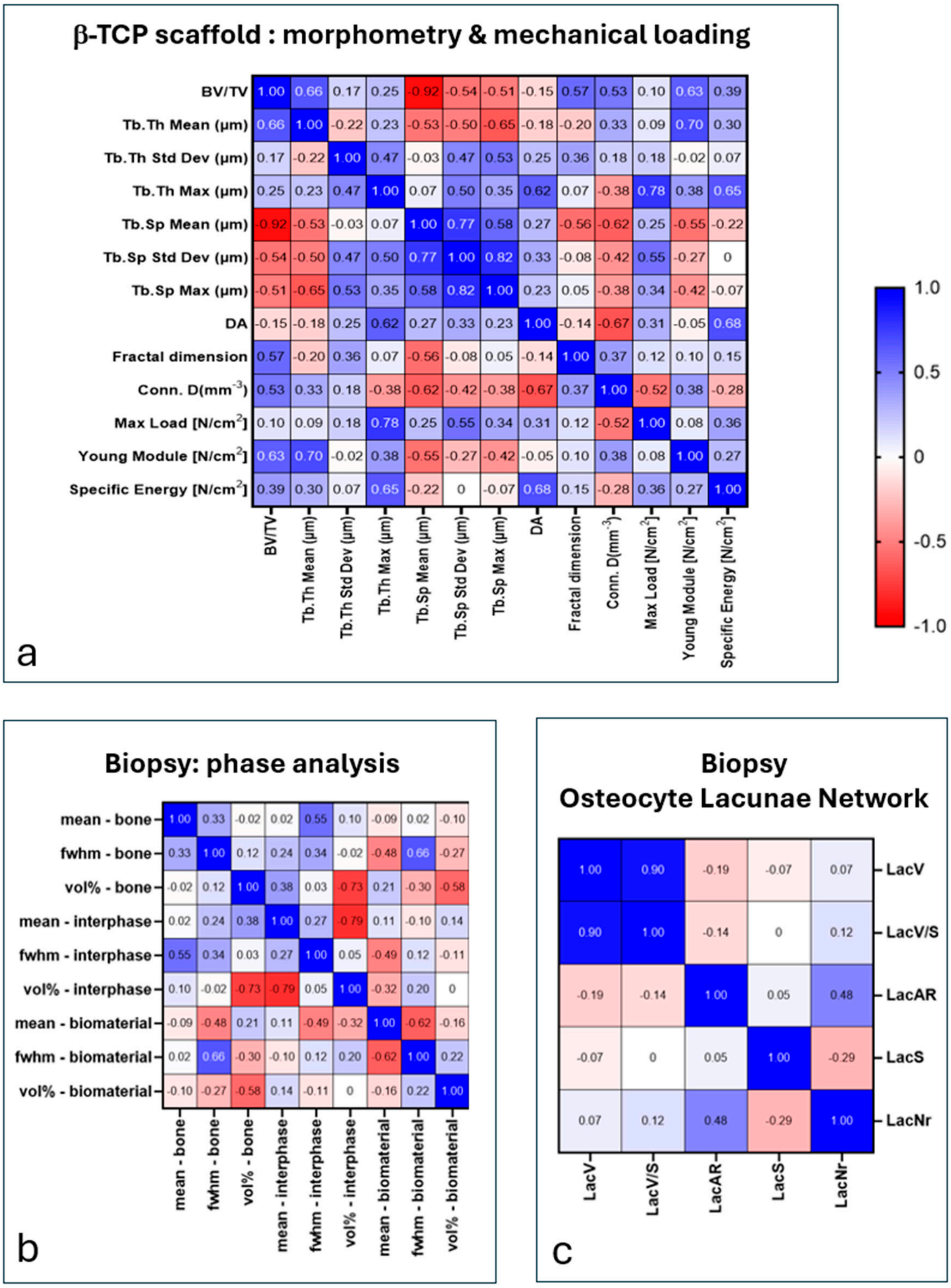

Figure S4. Absorbance IR spectra of β-TCP (light blue line), and bone hydroxyapatite (red line). The spectra are displayed in the1800-900 cm^{-1} spectral range; the position (in terms of wavenumbers, cm^{-1}) of the most significant peaks are indicated at the top.

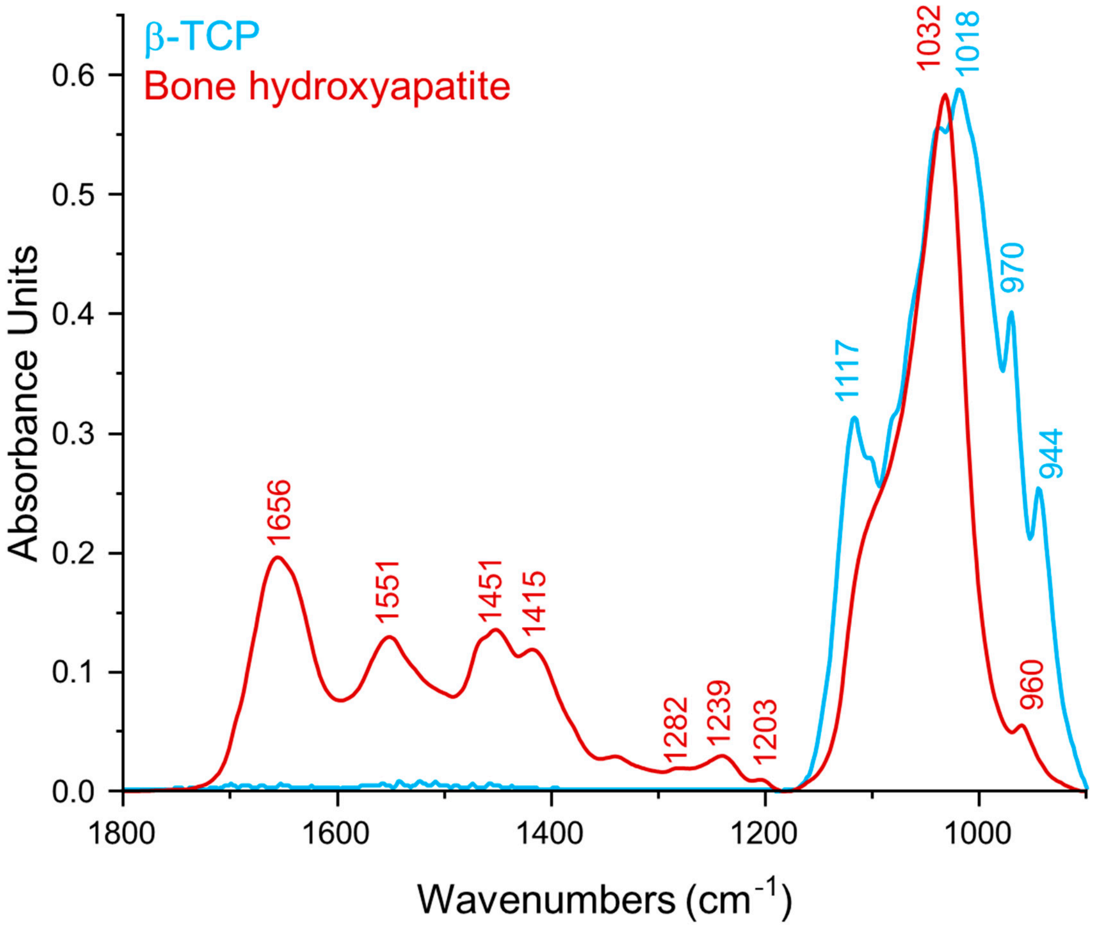

Table S3. Biomaterials performance in Sinus Lift Augmentation- Microarchitecture in Clinical cases.

Biomaterial	NB (vol.%)	ResBio (vol.%)	TB.Th (µm)	TB.Sp (µm)	Conn.D(mm-1)	FD	DA	t (mo.s)	Reference
Bio-Oss ®	37.2 (4.7)	36.6 (3.4)	-	-	-	-	-	6	DOI: 10.1111/cid.13331
Bio-Oss ® + Bio-Gide® collagen membrane	36.6 (3.4)	27.2 (5)	-	-	-	-	-	6	DOI: 10.1111/cid.13331
Bio-Oss ®	34.11		50	60	-	-	-	6	DOI: 10.3390/medicina60111834
Cerabone®	66.06%		80	40	-	-	-	6	DOI: 10.3390/medicina60111834
human bone allograft (MHBA)	37.2	19.55	-	-	-	-	-	5–13	DOI: 10.1111/clr.12225
platelet-rich fibrin (A-PRF) + serum albumin-coated bone allograft (SACBA)	21.2	0	184	520	1988	-	-	6	DOI: 10.3390/ma14071810
Autologous + Bio-Oss ®	48.2 (21.6–90.0)		234 (159–556)	186 (97–281)	-	-	0.139 (0.119–0.206)	6–8	DOI: 10.1111/clr.12380
Autologous + BoneCeramic®	43.2 (13.5–67.4)		185 (110–220)	195 (100–330)	-	-	0.127 (0.112–0.149)	6–8	DOI: 10.1111/clr.12380
Cortico-cancellous porcine bone (mp3®)	62.0(6.2)		105 (14)	64 (9)	-	-	-	6	DOI 10.1007/s00784-017-2139-6
Biphasic Calcium Phosphate (HA/b-TCP 30/70) - Blocks	18.9 (6.3)	27.3 (7.5)	50.3 (4.8)	-	-	-	0.617 (0.090)	5-6	DOI: 10.1097/ID.0000000000000363
Biphasic Calcium Phosphate Scaffolds (HA/b-TCP 30/70) - Granules	26.5 (8.9)	19.7 (3.6)	55.0 (3.5)	-	-	-	0.495 (0.158)	5-6	DOI: 10.1097/ID.0000000000000363
Pure Biocoral	30.5	1.9	69	144	-	-	0.19	6-7	DOI 10.1111/cid.12039
Biocoral + TCP	16.8	35.2	189	148	-	-	0.16	6-7	DOI 10.1111/cid.12039
Biphasic Calcium Phosphate (HA/b-TCP 30/70) - Blocks	26.0	2.9	55	137	-	-	0.13	6-7	DOI 10.1111/cid.12039

NB (vol.%): newly formed bone (volume percentage); ResBio (vol.%): residual biomaterial (volume percentage);TB.Th (µm): mean trabecular thickness; TB.Sp (µm): mean trabecular spacing; Conn.D (mm^{-1}): connectivity density; FD: fractal dimension; DA: anisotropy degree; t (mo.s): healing time (months).
